# Humanized bispecific antibody (mPEG × HER2) rapidly confers PEGylated nanoparticles tumor specificity for multimodality imaging in breast cancer

**DOI:** 10.1186/s12951-020-00680-9

**Published:** 2020-08-27

**Authors:** Yi-An Cheng, Tung-Ho Wu, Yun-Ming Wang, Tian-Lu Cheng, I-Ju Chen, Yun-Chi Lu, Kuo-Hsiang Chuang, Chih-Kuang Wang, Chiao-Yun Chen, Rui-An Lin, Huei-Jen Chen, Tzu-Yi Liao, En-Shuo Liu, Fang-Ming Chen

**Affiliations:** 1 Department of Biomedical Science and Environmental Biology, Kaohsiung Medical University, No.100, Shin-Chuan 1st Road, Sanmin Dist, Kaohsiung, Taiwan; 2 Cardiovascular Division of Surgical Department, Kaohsiung Veterans General Hospital, No.386, Dazhong 1st Rd, Zuoying Dist, Kaohsiung, Taiwan; 3 Department of Biological Science and Technology, Institute of Molecular Medicine and Bioengineering, National Chiao Tung University, No. 1001, University Road, Hsinchu, Taiwan; 4 Graduate Institute of Medicine, Kaohsiung Medical University, No.100, Shin-Chuan 1st Road, Sanmin Dist, Kaohsiung, Taiwan; 5 Department of Medical Research, Kaohsiung Medical University Hospital, No. 100, Tzyou 1st Road, Kaohsiung, Taiwan; 6 Graduate Institute of Pharmacognosy, Taipei Medical University, No.172-1, Sec. 2, Keelung Rd, Taipei, Taiwan; 7 Department of Radiology, Faculty of Medicine, College of Medicine, Kaohsiung Medical University, No.100, Shin-Chuan 1st Road, Sanmin Dist, Kaohsiung, Taiwan; 8 Department of Medical Imaging, Kaohsiung Medical University Hospital, No. 100, Tzyou 1st Road, Kaohsiung, Taiwan; 9 Division of Breast Surgery, Department of Surgery, Kaohsiung Medical University Hospital, No. 100, Tzyou 1st Road, Kaohsiung, Taiwan; 10 Department of Surgery, Kaohsiung Municipal Ta-Tung Hospital, No.68, Jhonghua 3rd Rd, Cianjin District, Kaohsiung, Taiwan; 11 Department of Surgery, Faculty of Medicine, College of Medicine, Kaohsiung Medical University, No.100, Shin-Chuan 1st Road, Sanmin Dist, Kaohsiung, Taiwan; 12 Drug Development and Value Creation Research Center, Kaohsiung Medical University, No.100, Shin-Chuan 1st Road, Sanmin Dist, Kaohsiung, Taiwan; 13 Department of Medicinal and Applied Chemistry, Kaohsiung Medical University, No.100, Shin-Chuan 1st Road, Sanmin Dist, Kaohsiung, Taiwan

**Keywords:** Bispecific antibody, PEGylated nanoparticle, Contrast agent, Multimodality image, Polyethylene glycol, Anti-PEG antibody, One-step formulation, Tumor specificity, Cancer image

## Abstract

**Background:**

Developing a universal strategy to improve the specificity and sensitivity of PEGylated nanoaparticles (PEG-NPs) for assisting in the diagnosis of tumors is important in multimodality imaging. Here, we developed the anti-methoxypolyethylene glycol (mPEG) bispecific antibody (BsAb; mPEG × HER2), which has dual specificity for mPEG and human epidermal growth factor receptor 2 (HER2), with a diverse array of PEG-NPs to confer nanoparticles with HER2 specificity and stronger intensity.

**Result:**

We used a one-step formulation to rapidly modify the nanoprobes with mPEG × HER2 and optimized the modified ratio of BsAbs on several PEG-NPs (Lipo-DiR, SPIO, Qdot and AuNP). The αHER2/PEG-NPs could specifically target MCF7/HER2 cells (HER2^++^) but not MCF7/neo1 cells (HER2^+/−^). The αHER2/Lipo-DiR and αHER2/SPIO could enhance the sensitivity of untargeted PEG-NPs on MCF7/HER2 (HER2^++^). In in vivo imaging, αHER2/Lipo-DiR and αHER2/SPIO increased the specific targeting and enhanced PEG-NPs accumulation at 175% and 187% on 24 h, respectively, in HER2-overexpressing tumors.

**Conclusion:**

mPEG × HER2, therefore, provided a simple one-step formulation to confer HER2-specific targeting and enhanced sensitivity and contrast intensity on HER2 positive tumors for multimodality imaging. 
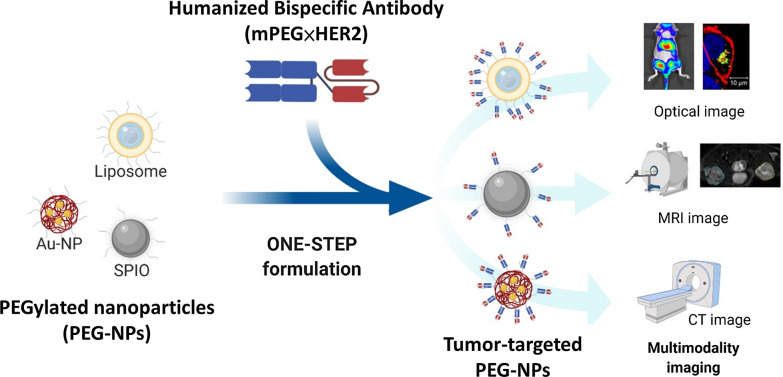

## Introduction

Non-invasive imaging for *in vivo* tracking of the location and size of tumors is very important in cancer therapy and diagnostics. Optical imaging (OI) is relatively inexpensive and robust for all kinds of molecular and cellular processes in small animals, but clinical applications are hindered by limited depth penetration [1]. Magnetic resonance imaging (MRI) has spectacular resolution and is particularly well suited for examining non-bony parts and soft tumors (e.g. breast, brain, etc.) in the clinic, but imaging sensitivity is inferior to nuclear techniques [2]. Nuclear imaging is characterized by high sensitivity, but suffers from poor spatial and temporal resolution [3]. Thus, development of multimodality imaging protocols can help overcome the limitations of single imaging modalities [4]. Many nano-contrast agents have been developed such as liposomes, microbubbles, superparamagnetic iron oxide (SPIO), AuNP and so on [5]. Most contrast agents are modified with methoxy-polyethylene glycol (mPEG) as PEG-NPs, which can enhance the biocompatibility and half-life of nanoparticles. However, the water-solubility of mPEG reduces the cell uptake of nanoparticles, thus, the PEG-NPs were reported to just passively accumulate in tumor site via the enhanced permeability and retention (EPR) effect that did not increase the cell uptake of nanoparticles in tumor cells [6], thereby limiting the sensitivity and signal intensity of PEG-NPs [7]. Therefore, active tumor-targeting and cell uptake of PEG-NPs is important to enhance the sensitivity for targeted diagnostics [8].

In order to provide tumor specificity to the PEG-NPs, the anti-tumor antibodies, ligands and peptides were conjugated with nanoprobes to form targeted contrast agents [9–12]. Freedman et al. showed that chemical conjugation of liposomal gadopentetate dimeglumine with anti-transferrin receptor scFv could increase the pixel intensity of small lung cancers (100 mm) in MRI images compared untargeted liposomes [8]. Chemical conjugation of anti-HER2/EGFR bispecific antibody to SPIO significantly enhanced the relative contrast enhancements in SKBR-3 tumors (HER2^+++^) as compared to colo-205 tumors (HER2^−^) at 24 h post-injection [13]. However, the chemical conjugation of the functional groups of antibodies to PEG-NPs caused antibody dysfunction, because the coupling site blocks the antigen-binding site of antibody and chemical reagents alter the protein structure. Protein adaptors, such as protein G, biotin and streptavidin, have been developed to non-covalently modify nanoparticles for stabilizing the structure of antibody. For example, streptavidin was used as an adaptor to connect the biotinylated anti-CD45RO antibody and biotinylated PEGylated lipid nanoparticles for selective targeting into memory T cells [14]. Protein G (IgG-binding b2 domain) was conjugated to gold nanoparticles with anti-HER2 antibody for specific targeting to HER2 overexpressing breast cancer [15]. Nevertheless, using exogenous adaptors, which induce immunogenicity, is not allowed in the human body, leading to reducing the half-life of PEG-NPs and limiting the rapid development of molecular imaging in clinic. Thus, developing a modification method which is simple, convenient and has low immunogenicity for universal contrast materials is important to improve the tumor specificity and sensitivity of targeted PEG-NPs.

**Fig. 1:**
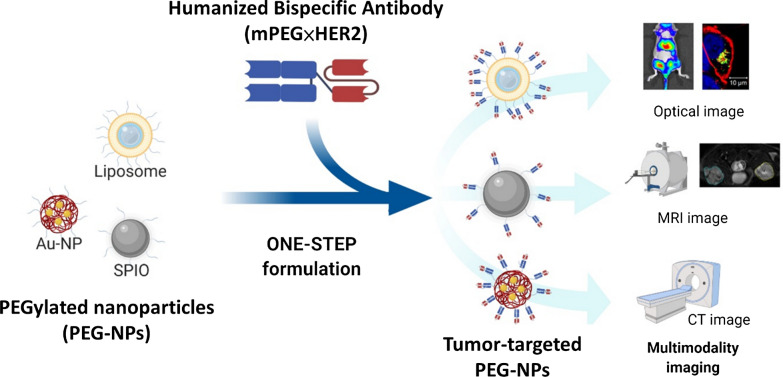
Humanized Bispecific Antibody (mPEG × HER2) Rapidly Confers PEG-NPs Tumor Specificity for Multimodality Imaging in Breast Cancer anti-mPEG BsAbs (mPEG × markers) provide an easy, universal and one-step formulation for any naive PEG-NPs to accelerate the development of targeted PEG-NPs for multimodality imaging in the clinic

We previously established humanized bispecific antibody (BsAb; mPEG × HER2) which can bind to the terminal methoxy groups present on PEG chains surrounding PEGylated drugs to confer HER2-binding specificity to nanoparticles. Humanized BsAbs can provide non-covalent modification as a simple one-step formulation on PEG-NPs [16]. In this study, we investigated whether multiple PEG-NPs (liposome, SPIO, Qdot and AuNP) could be modified by mPEG × HER2. Additionally, we examined the specific targeting and sensitivity of HER2-targeted nanoparticles in HER2 positive cancer cells using non-invasive imaging. For in vivo imaging, the signal intensity of HER2-targeted Lipo-DiR and SPIO were analyzed on HER2 positive tumors and HER2 negative tumors. This one-step formulation of PEG-NPs with mPEG × HER2 is a simple method to confer HER2-specific targeting and enhanced sensitivity and contrast intensity on HER2 positive tumors for multimodality diagnostic imaging (Fig. 1).

## Results

### One-step formulation of PEGylated nanoparticles with mPEG × HER2

**Fig. 2:**
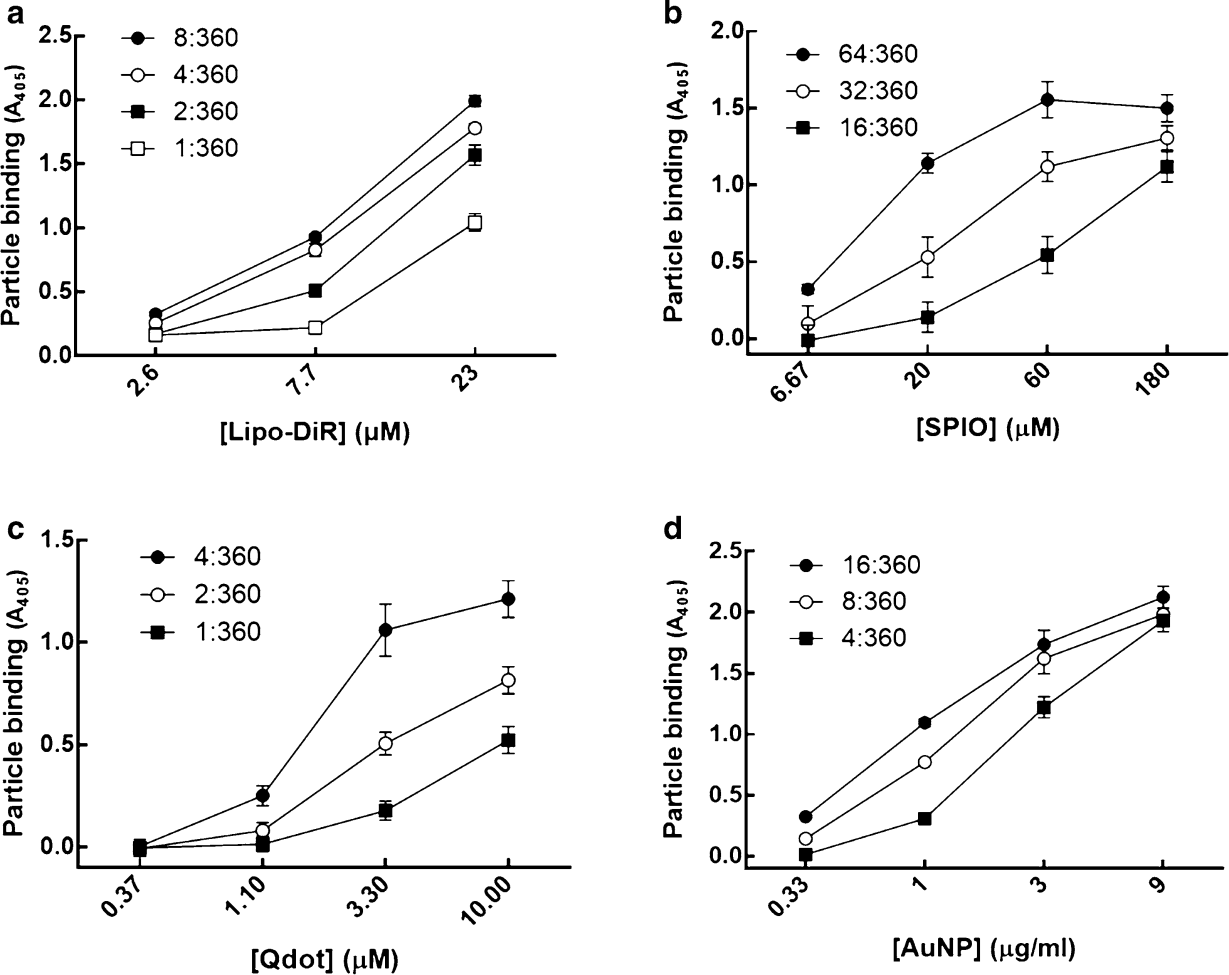
The HER2 binding ability of αHER2/PEG-NPs. Different BsAb:mPEG ratios of **(a)** αHER2/Lipo-DiR, **(b)** αHER2/SPIO, **(c)** αHER2/Qdot and **(d)** αHER2/AuNP were incubated with MCF7/HER2, and then anti-PEG antibody was added to detect PEG-NPs via ELISA (n = 3, triplicate). Bars, SD

To demonstrate whether the bispecific antibody (BsAb) could modify the PEG-NPs, we used mPEG × HER2 which was constructed from humanized bispecific antibodies by fusing the anti-mPEG Fab and anti-HER2 scFv via peptide linker (G4S)_3_, and the mPEG × DNS as a negative control which was created by exchanging the anti-HER2 scFv with an anti-DNS scFv, which binds the small chemical hapten dansyl. Frist, we quantified the mPEG concentration on PEG-NPs (Lipo-DiR, SPIO, Qdot and AuNP) by mPEG antibody-based sandwich ELISA [17], which uses the anti-PEG backbone antibody as a capture antibody and the anti-methoxy PEG antibody as a detection antibody. And then, different amounts of mPEG × HER2 were mixed with Lipo-DiR, SPIO, Qdot and AuNP, respectively, and the BsAb to mPEG modification ratio (BsAb:mPEG; mol:mol) was 64:360 to 1:360. The αHER2/PEG-NPs were quantified to the unconjugated BsAb to determine the BsAb conjugation rate of PEG-NPs. The αHER2/PEG-NPs were added to the mPEG-coated ELISA plates and then sequentially treated with anti-human Fab-HRP antibody and ABTS substrate to detect the unconjugated BsAbs. The BsAb conjugation ratio of αHER2/PEG-NPs was calculated by total number of BsAbs minus number of unconjugated BsAbs then divided by total number of BsAbs. The conjugation ratio of αHER2/PEG-NPs (Lipo-DiR, SPIO, Qdot and AuNP) was above 84.7 to 99% whereas Lipo-DiR and AuNP were observed in the precipitate in the highest BsAb:mPEG ratio group at 24 h incubation at 4 °C (Additional file 1: Table S1). We further compared the binding ability of the different BsAb:mPEG ratios of αHER2/PEG-NPs to MCF7/HER2 cells. The αHER2/PEG-NPs and αDNS/PEG-NPs (Lipo-DiR, SPIO, Qdot and AuNP) were incubated with MCF7/HER2 (HER2^++^) cells, and then the bound PEG-NPs were detected by using the anti-PEG backbone antibody. We chose the optimized BsAb:mPEG ratio of PEG-NPs, which had higher binding efficiency to HER2, of BsAb on Lipo-DiR, SPIO, Qdot and AuNP was 4:360, 64:360, 4:360 and 8:360, respectively (Fig. 2). We further checked the physical characteristics of αHER2/PEG-NPs and PEG-NPs (Lipo-DiR and SPIO) by dynamic light scattering. The particle size of αHER2/PEG-NP was slightly greater than that of PEG-NPs (Lipo-DiR: 96.6 nm versus 90.4 nm; SPIO: 100.5 nm versus 95.1 nm). The zeta potential of αHER2/PEG-NP was similar to that of PEG-NPs (Lipo-DiR: − 10.6 mA versus − 10.13 mA; SPIO: − 5.28 mA versus − 5.18 mA). The polydispersity index (PDI) values for all particles were around 0.1 (Additional file 1: Table S2). The result indicates that mPEG × HER2 conjugation did not alter the physical characteristics of PEG-NPs. The results indicated that the BsAb could modify the diverse PEG-NPs by a simple one-step method.

### The specificity of αHER2/PEG-NPs on HER2-overexpressing cells

**Fig. 3:**
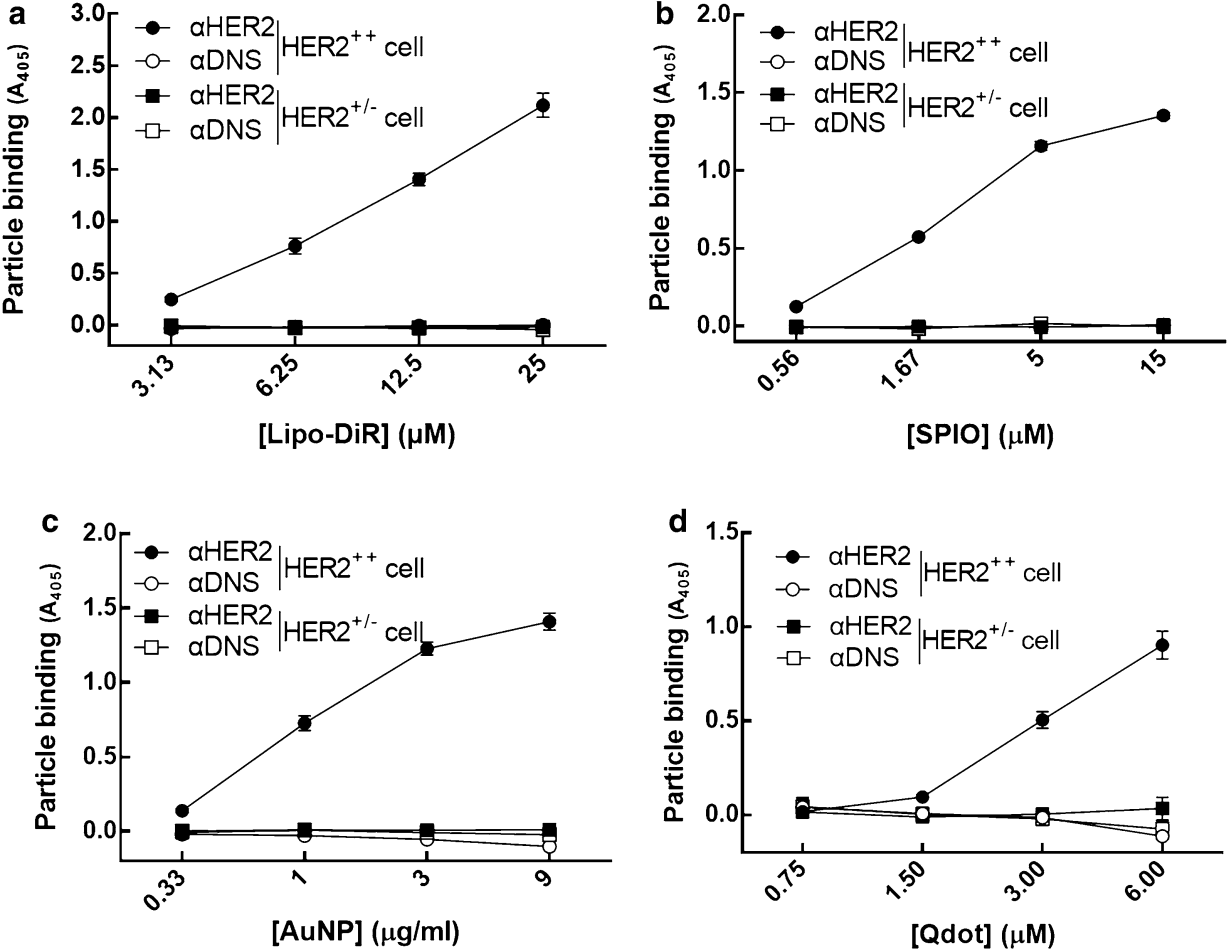
Specificity of αHER2/PEG-NPs for HER2^++^ cancer cells. MCF7/HER2 (HER2^++^) (circle shape) and MCF7/neo1(HER2^+/−^) (square shape) cancer cells in 96-well plates were incubated with mPEG × HER2 (solid shape) and mPEG × DNS (hollow shape) modified with different contrast agents. After washing, bound contrast agents were detected by ELISA (n = 3, triplicate). Bars, SD

To examine the tumor specificity of optimized targeted PEG-NPs, we used MCF7/HER2 cells which express high levels of HER2, and MCF7/neo1 cells with low levels of HER2. The αHER2/PEG-NPs and αDNS/PEG-NPs (Lipo-DiR, SPIO, Qdot and AuNP) were incubated with MCF7/HER2 (HER2^++^) cells or MCF7/neo1 (HER2^+/−^) cells, and then the mPEGs of PEG-NPs were detected by using the anti-PEG backbone antibody, anti-mouse IgG Fc-HRP and ABTS. As shown in Fig. 3, the absorbance value of αHER2/PEG-NPs (Lipo-DiR, SPIO, Qdot and AuNP) was gradually increased as compared with αDNS/PEG-NPs. We demonstrated that mPEG × HER2 could confer HER2 specificity to the diverse PEG-NPs by one-step formulation.

### The sensitivity of αHER2/PEG-NPs on HER2-expressing cells

**Fig. 4:**
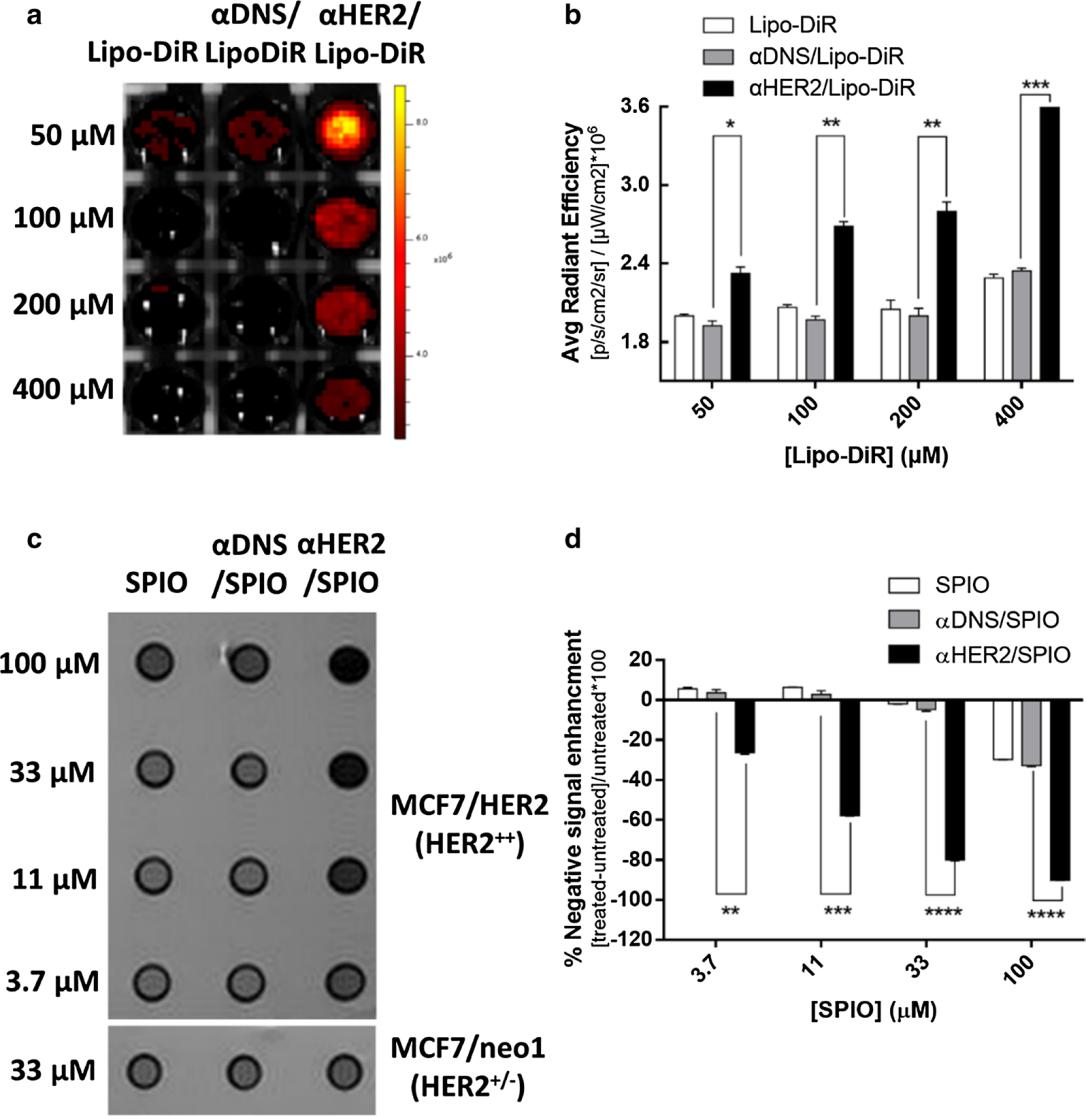
*In vitro* sensitivity image of αHER2/PEG-NPs. MCF7/HER2 (HER2^++^) cancer cells incubated with HER2 targeted-contrast agent with serial dilution concentrations. **a** αHER2/Lipo-DiR, αDNS/Lipo-DiR and Lipo-DiR were added to cells. Fluorescence images were obtained by the IVIS spectrum system. **b** Calculations of average radiant efficiency of (**a**). **c** αHER2/SPIO, αDNS/SPIO and SPIO were added to cells. MR imaging was performed with a 7.0 T MR imaging scanner. **d** The result from (**c**) was calculated by [treated SI-untreated SI]/untreated SI*100. *P < 0.05, **P < 0.01, ***P < 0.001, ****P < 0.0001 (unpaired t test)

We wanted to know whether the one-step formulation of PEG-NPs with mPEG × HER2 improves their sensitivity to HER2^++^ tumor cells. For optical imaging, we used Lipo-DiR as the near-infrared fluorescence liposome mixed with BsAbs. The various concentrations of αHER2/Lipo-DiR, αDNS/Lipo-DiR, or Lipo-DiR were incubated with MCF7/HER2, and then the fluorescence of DiR was detected by IVIS imaging. We calculated the relative fluorescence intensity by dividing the ROI of Lipo-DiR to the cells alone. As shown in Fig. 4a, the fluorescence intensity of αHER2/Lipo-DiR was significantly stronger than that of αDNS/Lipo-DiR, or Lipo-DiR. At high concentrations, the fluorescence intensity of αHER2/Lipo-DiR was 1.6 fold higher than Lipo-DiR. At low concentrations, αHER2/Lipo-DiR was still 1.2 fold higher than Lipo-DiR (Fig. 4b). For MR imaging, αHER2/SPIO, αDNS/SPIO or SPIO was incubated with MCF7/HER2 and the SPIO accumulation was examined by MRI. As shown in Fig. 4c, d, the strong MR signals as visualized by a darker color were only observed for αHER2/SPIO. After calculation, αHER2/SPIO still had 1.75 fold higher signal intensity (SI) at low concentration (1.5 μg/ml) whereas the signal of αDNS/SPIO and SPIO was undetectable. The results showed that the one-step formulation of mPEG × HER2 could enhance the image intensity of the PEG-NPs. Additionally, the detectable concentration of the αHER2/PEG-NPs is significantly lower than that of the untargeted PEG-NPs.

### The tumor delivery of αHER2/Lipo-DiR to HER2-expressing tumors

**Fig. 5:**
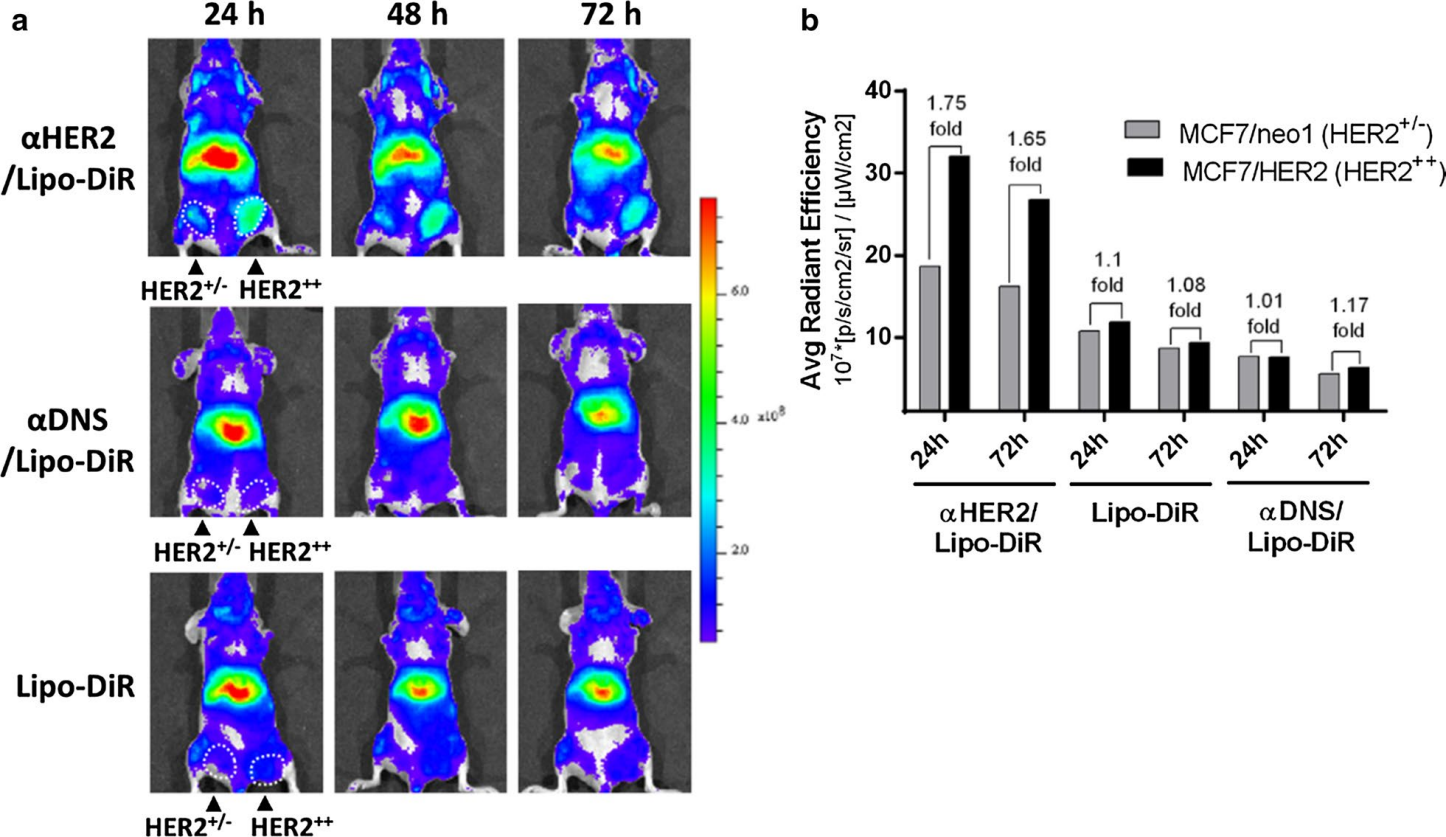
*In vivo* IVIS imaging of αHER2/Lipo-DiR and αDNS/Lipo-DiR. The delivery of αHER2/Lipo-DiR in HER2-overexpressing tumors. **a** αHER2/Lipo-DiR, Lipo-DiR and αDNS/Lipo-DiR were intravenously injected in mice bearing HER2^++^ (right m.f.p) and HER2^+/−^ (left m.f.p) tumors. The fluorescence intensity of DiR was detected at 24 h, 48 h and 72 h after injection by IVIS. The radiant efficiency of color bar was calculated by [p/s/cm^2^/sr]/[µW/cm^2^]. **b** Quantification of average radiant efficiency in HER2^++^ tumor and HER2^+/−^ tumor at 24 h and 72 h

To investigate whether mPEG × HER2 can enhance tumor accumulation of PEGylated liposomal DiR (Lipo-DiR) in HER2 over-expressing tumors, we first mixed mPEG × HER2 and mPEG × DNS with Lipo-DiR to form αHER2/Lipo-DiR and αDNS/Lipo-DiR. Mice bearing MCF7/HER2 (HER2^++^, in right m.f.p) and MCF7/neo1 (HER2^+/−^, in left m.f.p) tumors were intravenously injected with αHER2/Lipo-DiR, Lipo-DiR and αDNS/Lipo-DiR, and then the fluorescence of DiR was detected by IVIS imaging at 24 h, 48 h and 72 h. The fluorescence signal of αHER2/Lipo-DiR was enhanced in MCF7/HER2 (HER2^++^) tumors as compared to MCF7/neo1 (HER2^+/−^) tumors from 24 h to 72 h after probe injection (Fig. 5a). We quantified the relative region of interest (ROI) by the average ROI of MCF7/HER2 divided by that of MCF7/neo1. The relative ROI of αHER2/Lipo-DiR at 24 h was 1.75-fold (3.21 × 10^8^ versus 1.86 × 10^8^) whereas Lipo-DiR and αDNS/Lipo-DiR produced 1.1-fold (1.18 × 10^8^ versus 1.07 × 10^8^) and 1.01-fold (0.76 × 10^8^ versus 0.75 × 10^8^), respectively (Fig. 5b). Moreover, the ROI of αHER2/Lipo-DiR was higher than Lipo-DiR and αDNS/Lipo-DiR. The results indicate that mPEG × HER2 can enhance specific targeting and tumor accumulation of PEG-NPs in HER2-overexpressing tumors.

### Tumor accumulation and contrast intensity of αHER2/SPIO on HER2-expressing tumors

**Fig. 6:**
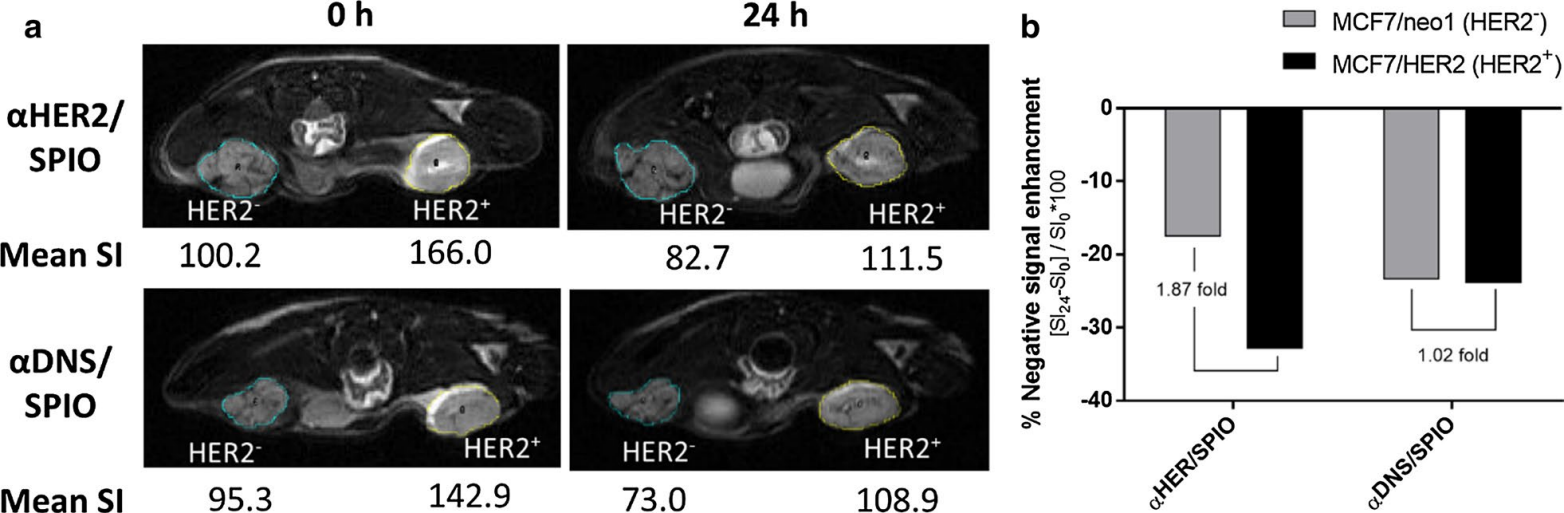
*In vivo* MR imaging of αHER2/SPIO and αDNS/SPIO. Nude mice bearing MCF7/HER2 (right m.f.p) and MCF7/neo1 (left m.f.p) tumors were intravenously injected with αHER2/SPIO and αDNS/SPIO (10 mg/kg). **a** Mice were sequentially imaged at pre-treatment and 24 h with a MR imaging scanner. **b** The result was calculated as the percentage of negative signal enhancement by [pretreated signal intensity (SI_0_)-treated signal intensity (SI_24_)]/SI_0 _* 100

To examine the in vivo tumor accumulation of αHER2/SPIO in HER2-positive tumors by MR imaging, αHER2/SPIO or αDNS/SPIO were intravenously injected into mice bearing subcutaneous MCF7/HER2(HER2^++^) tumors and MCF7/HER2(HER2^+/−^). The mean signal intensity (SI) was detected by T2-weighted fast spin-echo sequence 7.0 T imaging for every 3 mm sectioning thickness at 0 h and 24 h post-injection time. The percentage of negative contrast enhancement was calculated as the SI_24_ minus the SI_0_ divided by the SI_0_. The negative contrast enhancements of αHER2/SPIO were − 32.8% (MCF7/HER2) and − 17.5% (MCF7/neo1) whereas those of αDNS/SPIO were − 23.8% (MCF7/HER2) and − 23.3% (MCF7/neo1) (Fig. 6a). We calculated the relative contrast enhancements in MCF7/HER2 tumors as compared to MCF7/neo1. The relative contrast enhancements of αHER2/SPIO were 187%, higher than the 102% of αDNS/SPIO (Fig. 6b). The results indicate that mPEG × HER2 can enhance specific targeting and accumulation of PEG-NPs in HER2-overexpressing tumors.

## Discussion

We have successfully demonstrated that anti-mPEG BsAbs (mPEG × HER2) could be conjugated to PEG-NPs by a one-step process to confer HER2 specificity on MCF7/HER2 (HER2-expressing cells) for multimodality imaging. Without changing the structure of nanoparticles, the PEG-NPs (liposome, Qdot, AuNP and SPIO) were enhanced with HER2 targeting by modification at an optimized mPEG × HER2-conjugation ratio. In non-invasive imaging, αHER2/Lipo-DiR and αHER2/SPIO could enhance the intensity of the image signal on MCF7/HER2 tumors in MRI imaging, as compared with untargeted PEG-NPs. Due to the one-step modification, the anti-mPEG BsAb did not change the structure of the nanoparticles. This strategy can be easily applied to a diverse array of PEG-NPs for multimodality imaging. Moreover, the tumor specificity of anti-mPEG BsAbs can also be changed for different markers for corresponding diseases. The anti-mPEG BsAbs can confer tumor targeting to a variety of PEG-NPs. This technique can accelerate the development of targeted PEG-NPs to give more accurate diagnoses.

Developing a universal strategy to confer tumor specificity to each PEG-NPs is important for multimodality imaging. Thus, many studies have focused on developing bifunctional proteins as a universal antibody-conjugation method suitable for any PEG-NPs. For example, Hussain, et al. generated the EGFR × SNAP bifunctional protein by fusing anti-EGFR scFv and SNAP tag, which allowed covalent attachment of O^6^-benzylguanine (BG) modified dendritic polyglycerol doxorubicin conjugates (PG-Doxo) via the disulfide bond. The anti-EGFR-PG-Doxo resulted in a tenfold increase of uptake into EGFR-positive cancer cells compared with untargeted PG-Doxo [18]. Moreover, Schneider et al. developed VEGFR2 × Dig bispecific antibodies, which endowed the digoxigenin-modified CD31 siRNA liposome (Dig-LNP-siCD31) with VEGFR2 specificity, and anti-VEGFR2-LNP-siCD3 decreased the expression of CD31 RNA about two-fold compared with LNP-siCD31 alone in MCF-7 tumors [19]. However, BG and Dig haptens are not approved for human use by the FDA, leading to a limit in the clinical application of targeted PEG-NPs. We developed the universal bispecific antibody (mPEG × HER2), which can directly modify naïve PEG-NPs and simultaneously confer HER2 targeting. Methoxy-PEG hapten has been approved by the FDA for human use to modify nanoprobes, such as SPIO, AuNP and microbubbles, to improve the biocompatibility and half-life of PEG-NPs in vivo. Our results showed that a one-step formulation of mPEG × HER2 BsAbs with multiple PEG-NPs (liposome, Qdot, AuNP and SPIO) could enhance the HER2 targeting ability of PEG-NPs to HER2-positive tumor cells compared with untargeted PEG-NPs. Thus, anti-mPEG BsAbs provide an easy, universal and direct formulation for any naive PEG-NPs without chemical modification which can accelerate the development of targeted PEG-NPs for multimodality imaging in the clinic.

Developing an easy method to produce targeted PEG-NPs against the various disease-associated markers is important for imaging. Chemical modification of antibodies on nanoparticles is currently a common method to confer targeting of a disease [20, 21]. For example, Paulis et al. coupled the anti-ICAM-1antibody to N-succinimidyl S-acetylthioacetate (SATA) to generate a free thiol group for conjugating with the maleimide-PEG-liposomal MRI contrast agent (L), and they proved that the binding of anti-ICAM-1 L on bEnd.5 endothelial cells could be twofold higher than that of L to monitor the inflammation-related ICAM-1expression on blood vessels [22]. Chen, et al. generated anti-HER2 scFv containing the azide group for conjugating with the amine group of Cy5-dots to form anti-HER2 Cy5-dots, and the tumor uptake of anti-HER2 Cy5-dots had a 2.4 fold increase compared with untargeted dots in BT474 breast tumor-bearing mice [23]. However, the chemical conjugations of the antibody were random and multi-step leading to heterogeneous orientations of antibody. Furthermore, the process was time consuming [24]. Additionally, the chemical method is not universal and needs to be redesigned for different PEG-NPs. Our strategy used mPEG × HER2 to confer PEG-NPs (Lipo-DiR and SPIO) with HER2 specificity by one-step modification. Compared with untargeted nanoprobes, the cell imaging showed that the contrast intensity of αHER2/Lipo-DiR and αHER2/SPIO was increased 1.6-fold and 1.75 fold on MCF7-HER2 cells, respectively. In addition, we demonstrated that αHER2/Lipo-DiR and αHER2/SPIO enhances accumulation in MCF7-HER2 tumors (HER2^++^) by 175% and 186% in mice compared with MCF7/neo1 tumor (HER2^+/−^). Moreover, the HER2 portion of the mPEG × HER2 can be changed to other disease-associated markers such as EGFR, PSMA, CD20, TfR for development of varied mPEG × marker BsAbs to easily confer PEG-NPs with different specificity. Therefore, mPEG × markers may provide an easy method to change the specificity of targeted PEG-NPs for imaging of various diseases.

Developing a tumor-targeted contrast agent with low immunogenicity is important to allow repeat administration of the probe in humans. Protein linkers, such as streptavidin–biotin or protein A, are commonly used to provide non-covalent modification to conjugate the antibodies on PEG-NPs. Jin et al. conjugated protein A on quantum dots to attach anti-CXCR4 antibody for tracking the location of CXCR4 receptors in living cells [25]. However, protein A is an exogenous protein from bacteria that may cause a risk of immunogenicity [26]. Paganelli et al. reported streptavidin as a linker to link the biotinylated αCEA antibody and biotinylated radioactive ^111^In tracker to increase the tumor accumulation of ^111^In-αCEA antibody in CEA-positive patients, but 63% of patients had the anti-streptavidin antibody [27]. The study indicated that exogenous proteins may cause the immunogenicity in humans, limiting the application of targeted PEG-NPs in the clinic [28, 29]. Humanization of antibodies has been approved by FDA to reduce the immunogenicity of antibodies from non-human species, and one-half (38/78) of antibodies in clinical use are humanized [30, 31]. To analyze the immunogenicity of humanized BsAb, we co-cultured the dendritic cells differentiated from human PBMCs with autologous CD4 + T cells and stimulated with mPEG × HER2 and mPEG × DNS, respectively, for 5 days. Then, we detected the proliferation of CD4^+^ T cells by ATPlite assay (Additional file 1: Figure S1). The result indicated that there was no significant difference of CD4 + T cell proliferation between mPEG × HER2–treated, mPEG × DNS-treated, and control group, indicating the humanized BsAbs may be expected to have low immunogenicity. This result corresponds with other studies that the humanization of antibodies can reduce the immunogenicity of antibodies from non-human species [31, 32]. Harding et al. demonstrated that comparing with the chimeric anti-EGFR antibody, the humanized anti-EGFR antibody could reduce the proliferation of CD4^+^ helper T cells [33]. Thus, the humanized anti-mPEG BsAbs may have low immunogenicity and be suitable for wide use in clinical imaging. Moreover, PEGylation of PEG-NPs could also reduce the immunogenicity of the contrast agent to prolong the half-life of contrast agents in the human body. Thus, the low immunogenicity of humanized BsAbs can confer PEG-NPs with tumor specificity by a one-step formulation and allow repeat administration of probes in humans.

## Conclusions

mPEG × HER2 provided a simple one-step method to conjugate PEG-NPs to confer HER2-specific targeting and enhanced sensitivity and contrast intensity on HER2 positive tumors for multimodality imaging. The BsAbs described here possess potential advantages for targeted imaging including: (i) anti-mPEG BsAbs could one-step modify PEG-NPs with homogeneous coupling orientations to enhance the specificity and sensitivity of imaging probes. (ii) The changeable properties and universal applicability of BsAbs can direct diverse PEG-NPs to different biomarkers expressed in various diseases for diagnosis. (iii) The humanized anti-mPEG BsAbs with low immunogenicity are suitable for direct human use. We believe that the one-step formulation of PEG-NPs with anti-mPEG BsAbs could accelerate the targeted imaging development to provide the accurate diagnoses in the clinic.

## Materials and methods

### PEGylated nanoparticles

PEGylated DOPC/CHOL Liposomes labeled with DiR (Lipo-DiR) were purchased from FormuMax Scientific (Sunnyvale, CA, USA). Superparamagnetic iron oxide (SPIO, MnMEIO- mPEG NPs) was from Prof. Yun-Ming Wang (National Chiao Tung University, Hsinchu, Taiwan). Qtracker 655 non-targeted quantum dots (Qdot) were purchased from Thermo Fisher Scientific (Waltham, USA). Gold nanoparticles (AuNP, AuNCs-PLGA-mPEG) were from Prof. Chih-Kuang Wang (Kaohsiung Medical University, Kaohsiung, Taiwan).

### Cells and animals

MCF7/HER2 and MCF7/neo1 human breast adenocarcinoma cells line were grown in Dulbecco’s modified Eagle’s medium/nutrient mixture F-12 (DMEM/F12, Thermo Fisher Scientific, Roskilde-Denmark) supplemented with 10% (vol/vol) fetal bovine serum (FBS, Thermo, Waltham, MA, USA), 1% (vol/vol) penicillin/streptomycin (Invitrogen, Carlsbad, CA) at 37 ℃ in an atmosphere of 5% (vol/vol) CO_2_ in air. Three to four-week-old BALB/cAnN.Cg-Foxn1nu/CrlNarl nude mice were purchased from the National Laboratory Animal Center, Taipei, Taiwan. Animal experiments were performed in accordance with institute guidelines.

### Bispecific antibodies and antibodies

Human bispecific antibodies were created by linking the C-terminus of an anti-methoxy PEG Fab (clone h15-2b [34]) to an anti-HER2 scFv or anti-DNS scFv via a flexible peptide (GGGGS)3 to form mPEG × HER2 and mPEG × DNS, respectively. The anti-HER2 scFv was constructed by linking the 4D5 VH and VL domains with a linker (GGGGS)_3_; the detailed description of BsAbs was as described in a previous study [16]. The VL-Cκ and VH-CH1-linker-scFv domains were separated with an IRES in the pLNCX retroviral vector (BD Biosciences, San Diego, CA) in the unique Hind III and Cla I restriction enzyme sites to generate pLNCX-mPEG × HER2 and pLNCX-mPEG × DNS plasmids. Expi-293 cells were transfected with plasmids and the culture medium was collected after five days. The BsAbs were purified by affinity chromatography on gel prepared by reacting 36 mg of O-(2-Aminoethyl)-O’olyethylene glycol 750 (Sigma Aldrich) per gram of CNBr-activated Sepharose 4B (GE Healthcare, Little Chalfont, UK).

### Bi-functional assay of mPEG × HER2 and mPEG × DNS

Ninety-six well plates were coated with 50 μg/ml of poly-d-lysine in PBS for 5 min at 37 ℃, washed twice with deuterium depleted water and then coated with 2 × 10^5^ cells/well of MCF7/HER2 (HER2^++^) cancer cells. To fix cells, paraformaldehyde (2%, vol/vol) was added, left for 5 min then neutralized by 0.1 M glycine. mPEG × HER2 or mPEG × DNS (10 μg/ml) were added to the wells at room temperature for 30 min. After extensive washing, 10 μg/ml of mPEG2K-BSA was added to the wells for 30 min. After extensive washing, the bound concentrations of mPEG2K-BSA were determined by adding 10 μg/ml of 6–3 anti-PEG backbone antibody for 30 min and then adding 0.4 μg/ml of goat anti-mouse IgG Fc-HRP (Jackson ImmunoResearch Laboratories). The wells were washed and then ABTS substrate was added for 30 min before absorbance values at 405 nm were measured in a microplate reader (Biochrom, St Albans, United Kingdom).

### One-step formulation of PEG-NPs with BsAbs

PEG-NPs (Lipo-DiR, SPIO, Qdot and AuNP) were mixed with mPEG × HER2 in PBS at 4 °C for 5 min to form αHER2/PEG-NPs, respectively. The BsAb:mPEG molar ratios of αHER2/PEG-NPs was 64:360 with two-fold serial dilution to 1:360. To quantify the unconjugatd BsAb in αHER2/PEG-NPs, the particles with different BsAb:mPEG ratios were incubated in mPEG2K-BSA coated 96-well plates at RT for 45 min. After extensive washing, the BsAb was detected by 0.4 μg/ml of goat anti-human Fab-HRP, and then ABTS substrate was added for 30 min before absorbance values at 405 nm were measured by EZ Read 400 ELISA. The BsAb-conjugation rate of αHER2/PEG-NPs was calculated as the total number of BsAb minus number of unconjugated BsAb, then divided by the total number of BsAb.

### Specificity of αHER2/PEG-NPs for HER2^++^ cells

To examine the ability of PEG-NPs (Lipo-DiR, SPIO, Qdot and AuNP) modified with various ratios of mPEG × HER2 to bind to cancer cells expressing HER2, MCF7/HER2 cells and low HER2 expression cells, MCF7/neo1 (2 × 10^5^ cell/well) were seeded in poly-d-lysine-coated ninety-six well plates overnight at 37 °C. After fixing the cells, αHER2/PEG-NPs made with the various densities of BsAb on PEG-NPs were added to the wells at RT for 20 min. After extensive washing with PBS, the bound concentrations of PEG-NPs were determined by sequentially adding 10 μg/mL of 6-3 anti-PEG antibody for 1 h, washing with DMEM three times, and then adding 0.4 μg/mL of goat anti-mouse IgG Fc-HRP. The wells were washed three times with PBS and then ABTS substrate was added for 30 min before absorbance values at 405 nm were measured in EZ Read 400 ELISA. To further analyze HER2 specific targeting efficacy of optimized BsAb-modified PEG-NPs (Lipo-DiR, SPIO, Qdot and AuNP), serial dilutions of αHER2/PEG-NPs and αDNS/PEG-NPs were incubated with MCF7/HER2 cells in poly-d-lysine coated 96-well plates. PEG-NPs binding was measured as described above.

### Fluorescence imaging of αHER2/Lipo-DiR in vitro and in vivo

MCF7/HER2 (5 × 10^6^/well) cells were incubated with αHER2/Lipo-DiR, αDNS/Lipo-DiR or Lipo-DiR. After washing with PBS three times, cells were imaged with an IVIS spectrum optical imaging system (excitation, 750 nm; emission, 780 nm; PerkineElmer, Waltham, MA). BALB/c nude mice bearing MCF7/HER2 (HER2^++^) and MCF7/neo1 (HER2^+/−^) tumors (~ 100 mm^3^) in the mammary fat pad (m.f.p) were intravenously injected with αHER2/Lipo-DiR and Lipo-DiR (DiR concentration: 10 nmole per mouse), respectively. The mice were imaged on an IVIS spectrum optical imaging system at 24, 48 h and 72 h after injection. The regions-of-interest (ROI) in the tumor areas were drawn and analyzed with Living Image software version 4.2 (Caliper Life Sciences); radiant efficiency was calculated by [p/s/cm^2^/sr]/[µW/cm^2^].

### MR imaging of αHER2/SPIO in vitro and in vivo

MCF7/HER2 cells (5 × 10^6^ cell/well) were incubated αHER2/SPIO, αDNS/SPIO or SPIO were added to the tubes and incubated at 37 °C for 1 h. After washing with PBS three times, cells precipitated at the bottom of the Eppendorf tube and were then imaged with 7T MRI (7T PharmaScan, Bruker) TR/TE, 3000/65 ms; echo train length, 10; flip angle, 150^o^; field of view, 6 cm × 6 cm; slice thickness, 1 mm; interslice gap, 0.1 mm (10% of slice thickness); and matrix, 192 × 192. BALB/c nude mice bearing MCF7/HER2 (HER2^++^) and MCF7/neo1 (HER2^+/−^) tumor (~ 100 mm^3^) were injected intravenously with αHER2/SPIO and αDNS/SPIO (10 mg/kg per mouse) in their mammary fat pad regions, respectively. Isoflurane anesthetized mice were imaged with 7T MRI at 0, 24 h after injection. TR/TE, 3000/65 ms; echo train length, 10; flip angle, 150; field of view, 4 cm; slice thickness, 1.2 mm; interslice gap, 0.12 mm (10% of slice thickness); and matrix, 256 × 210. The negative enhancement was calculated by [pretreated signal intensity (SI_0_)-treated signal intensity (SI_24_)]/SI_0_*100.

### Immunogenicity of BsAb

To prepare monocyte-derived dendritic cells (DCs), peripheral blood mononuclear cells (PBMCs) from healthy donor blood isolated by Ficoll-Paque and monocytes isolated using Miltenyi Pan Monocyte Isolation Kits and LS columns (Miltenyi Biotech). Monocytes were resuspended in RPMI1640 supplemented with 10% FCS, 2 mM l-glutamine, 100 units/ml penicillin/streptomycin, 2.5 μg/ml Fungizone and 500 units/ml recombinant human IL-4 (Invitrogen) and 500 units/ml recombinant human granulocyte–macrophage colony-stimulating factor (R&D systems), and then seeded 4 × 10^5^ cells/well in 24-well plate. On day 6, 20 ng/ml recombinant human TNF-α (Sigma) and 10 ng/ml IL-1β (R&D systems) were added to the cells to activate DCs for 24 h. On day 7, the harvested DCs were counted and then incubated 50 μg/ml mitomycin C for 30 min at 37 °C at a density of 1 × 10^6^ cells/ml, then washed extensively. Autologous CD4^+^ T cells were isolated on Day 7 by negative selection using CD4^+^ T Cell Isolation Kit II and LS columns (Miltenyi Biotech). After counting, 2 × 10^5^ CD4^+^ T Cells were added to 2 × 10^4^ mitomycin C-treated DCs and incubated with mPEG × HER2, mPEG × DNS at concentration of 350 nM in 96-well round bottom plates. Controls included dendritic cells plus CD4^+^ T cells alone and with concentration of 10 μg/ml phytohemagglutinin (PHA). Cells were cultured at 37 °C for 5 days. Proliferation was assessed by ATPlite Luminescence Assay kit (Perkin Elmer). Counts per minute (cpm) for each well were determined by multimode plate reader (Perkin Elmer).

## Supplementary information

**Additional file 1: Table S1.** The BsAb-conjugation rate of αHER2/PEG-NPs. **Table S2.** The Characterization of BsAb/mPEG-NPs. **Figure S1.** Immunogenicity of humanized BsAbs. We cocultured dendritic cells differentiated from human PBMCs with autologous CD4^+^ T cells and stimulated with control medium (represented as DC+T), PHA (as positive control), PEG×HER2, PEG×DNS, respectively, for 5 days. Then, we detected the proliferation of CD4+ T cells by ATPlite assay. Bars, SD. CPM, counts per minute; PBMC, peripheral blood mononuclear cell; PHA, phytohemagglutinin.

## Data Availability

All data generated or analyzed during this study are included in this published article.

## Abbreviations

PEG: Polyethylene glycol
mPEG: Methoxypolyethylene glycol
HER2: Human epidermal growth factor receptor 2
SPIO: Superparamagnetic iron oxide
Qdot: Quantum dots
AuNP: Gold nanoparticle
MRI: Magnetic resonance imaging
EPR: Enhanced permeability and retention
EGFR: Human epidermal growth factor receptor
BsAb: Bispecific antibody
DNS: Dansyl
ELISA: Enzyme-linked immunosorbent assay
Fab: Antigen-binding fragment
scFv: Single-chain variable fragment
HRP: Horseradish peroxidase
ABTS: 2,2′-azino-bis(3-ethylbenzothiazoline-6-sulfonic acid
SD: Standard deviation
ROI: Region of interest
SI: Signal intensity
IVIS: In vivo imaging systems
VEGFR2: Vascular endothelial growth factor 2
Dig: Digoxigenin
FDA: Food and drug administration
ICAM-1: Intercellular adhesion molecule 1
PSMA: Prostate-specific membrane antigen
TfR: Transferrin receptor
CXCR4: C-X-C motif chemokine receptor 4
CEA: Carcinoembryonic antigen
