# Supplementary material for: Humanized bispecific antibody (mPEG × HER2) rapidly confers PEGylated nanoparticles tumor specificity for multimodality imaging in breast cancer
Source: J Nanobiotechnology. 2020 Aug 27;18:118. doi: 10.1186/s12951-020-00680-9 (PMC7457265; doi:10.1186/s12951-020-00680-9)
Supplement: Supplementary file 1 — Additional file 1: Table S1. The BsAb-conjugation rate of αHER2/PEG-NPs. Table S2. The Characterization of BsAb/mPEG-NPs. Figure S1. Immunogenicity of humanized BsAbs. We cocultured dendritic cells differentiated from human PBMCs with autologous CD4+ T cells and stimulated with control medium (represented as DC+T), PHA (as positive control), PEG×HER2, PEG×DNS, respectively, for 5 days. Then, we detected the proliferation of CD4+ T cells by ATPlite assay. Bars, SD. CPM, counts per minute; PBMC, peripheral blood mononuclear cell; PHA, phytohemagglutinin. [file 12951_2020_680_MOESM1_ESM.docx]

**Title:**

**Humanized Bispecific Antibody (mPEG×HER2) Rapidly Confers PEGylated Nanoparticles Tumor Specificity for Multimodality Imaging in Breast Cancer**

Yi-An Cheng#, Tung-Ho Wu#, Yun-Ming Wang, Tian-Lu Cheng, I-Ju Chen, Yun-Chi Lu1, Kuo-Hsiang Chuang, Chih-Kuang Wang, Chiao-Yun Chen, Rui-An Lin, Huei-Jen Chen, Tzu-Yi Liao, En-Shuo Liu, Fang-Ming Chen*

**Additional file 1**

**Table S1. The BsAb-conjugation rate of αHER2/PEG-NPs**

|  | **BsAb:mPEG molar ratio** | | | | | | |
| --- | --- | --- | --- | --- | --- | --- | --- |
| PEG-NPs | 64:360 | 32:360 | 16:360 | 8:360 | 4:360 | 2:360 | 1:360 |
| Lipo-DiR | P | P | P | 94.6%, P | 94.9% | >99%* | >99%* |
| SPIO | 98.6% | 98% | >99%* | >99%* | N | N | N |
| Qdot | N | N | N | N | 90% | 91.2% | >99%* |
| Au-NP | N | 90.8%, P | 84.7% | 88.9% | N | N | N |

P: Observing the precipitate within 1 hour at 4^o^C

N: No result

*: Undetectable of unconjugated BsAb

**Table S2. The Characterization of BsAb/mPEG-NPs**

|  | **αHER2/Lipo-DiR** | **Lipo-DiR** | **αHER2/SPIO** | **SPIO** |
| --- | --- | --- | --- | --- |
| **Particle size (nm)** | 96.6 ± 17.2 | 90.4 ± 19.8 | 100.5 ± 72.7 | 95.1 ± 61.2 |
| **PDI*** | 0.131 | 0.102 | 0.089 | 0.13 |
| **Zeta potential** | -10.6 | -10.13 | -5.28 | -5.18 |

***PDI:** polydispersity index

Particle size data were expressed as mean ± SD (n = 3).


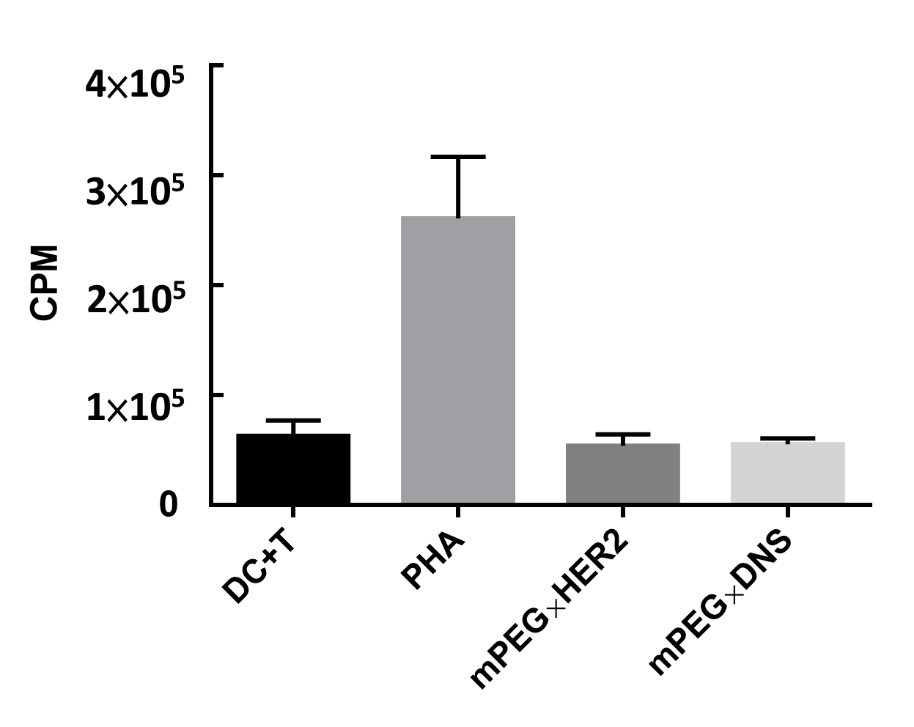


**Figure S1. Immunogenicity of humanized BsAbs**

We cocultured dendritic cells differentiated from human PBMCs with autologous CD4^+^ T cells and stimulated with control medium (represented as DC+T), PHA (as positive control), PEG×HER2, PEG×DNS, respectively, for 5 days. Then, we detected the proliferation of CD4+ T cells by ATPlite assay. Bars, SD. CPM, counts per minute; PBMC, peripheral blood mononuclear cell; PHA, phytohemagglutinin.
